# Training in robotic surgery, replicating the airline industry. How far have we come?

**DOI:** 10.1007/s00345-019-02976-4

**Published:** 2019-10-17

**Authors:** Justin William Collins, Pawel Wisz

**Affiliations:** 1 Department of Molecular Medicine and Surgery, Karolinska Institutet, Stockholm, Sweden; 2 Orsi Academy, Melle, Belgium; 3 OLV Hospital, Aalst, Belgium; 4 Department of Uro-oncology, UCLH (University College London Hospital), London, UK

**Keywords:** Robotic-assisted surgery, Training, Non-technical skills, Surgical education, Patient safety, Proficiency-based progression

## Abstract

**Introduction:**

As the role of robot-assisted surgery continues to expand, development of standardised and validated training programmes is becoming increasingly important. We aim to compare current robotic training curricula with training in aviation, to evaluate current similarities and to provide insight into how healthcare can further learn from replicating initiatives in aviation training.

**Methods:**

A systematic literature review of the current evidence was conducted online and relevant publications and information were identified. Evaluation and comparison between training in robotic surgery and the aviation industry was performed.

**Results:**

There are significant similarities between modern robotic training curricula and pilot training. Both undergo basic training before proceeding to advanced training. Aviation training methods include classroom instruction, e-learning and practical training, in both the aircraft and flight simulation training devices. Both surgeon and pilot training include technical and procedural instruction as well as training in non-technical skills such as crisis management, decision making, leadership and communication. However, there is more regulation in aviation, with international standards for training curricula, simulation devices and instructors/trainers that are legally binding. Continuous learning with re-qualification with benchmarked high stakes tests are also mandatory throughout a pilot’s and instructor’s career.

**Conclusion:**

Robotic surgeons and pilots roles have many fundamental similarities. Both work with expensive and complex technology requiring high levels of skills, within working environments with high physiological and psychological stress levels. Whilst many initiatives in aviation training have already been replicated in surgical training there remain considerable differences in regulation. Adopting established and proven aviation methods of assessment and regulation could help robotic surgical training become more efficient, more effective and ultimately safer.

## Introduction

The airline pilot training model has often been used as an analogy for improved surgical training [1]. For pilots, training methods include classroom instruction, e-learning and practical training, in both the aircraft and with flight simulation training. Pilot training focuses not only on technical and procedural instruction, but also training in non-technical skills, including crisis (emergency) management, decision making, leadership and communication, that benefits the team performance. Airline pilot training has many similarities with robotic surgery training with both having basic training before proceeding to advanced training. In robotic surgery advanced training relates to specialty-specific procedural training and for pilots, it relates to a specific aircraft type. In the near future, when multiple robotic surgical platforms become available, the need to ‘retrain’ in platform (device)-specific training modules will also become a further area of commonality.

The structure to training in aviation and robotic surgery has many similarities and both parties control complex technology with their hands, that if managed inappropriately could result in fatal consequences. However, the safety outcome records compared between the healthcare and aviation industries are very different. In the book ‘Why Hospitals should fly’, the author J Nance reported mortality comparisons between the aviation and healthcare industries over a 5-year period between 2001 and 2006, with zero deaths on commercial US flights compared to an estimated 250–500,000 deaths from medical errors in the US healthcare system at the same time [2].

In the United States between 2000 and 2013, 10,624 adverse events related to robotic procedures were reported [3]. In 2013, a group of experts expressed concern that robotic surgery training is random and insufficient to ensure patient safety [4]. Two years later the Emergency Care Research Institute (ECRI) institute on health technology hazards published an independent review in which a lack of robotic surgical training as 1 of the top 10 risks to patients was identified [5].

An important aspect that differs between aviation and surgical training is the increased standardisation with internationally agreed training standards in the airline industry. Evaluation and regulation comprise of benchmarked high stakes tests related to proficiency-based training that results in quality assurance [6]. Aviation training curricula, simulation training devices and instructors/trainers are standardised throughout the world and these standards are legally binding. Re-qualification and recurrent training to defined benchmarked metrics of performance are mandatory at all stages of a pilot’s and instructor’s career.

A second aspect that differs between the two industries is the focus on ‘systems thinking’ compared to focusing on individuals. System thinking addresses the ‘system’ or organisation as a whole and is key to the success of high-reliability organisations (HRO), such as aircraft carriers. Similarly, to the aviation industry, there are multiple factors involved in robotic surgical training that increase risks to patient safety, these include patient factors, provider factors, task factors, technology and tool factors, team factors, environmental factors and organisational factors. Surgery has multiple safety variables to manage simultaneously within an organisation and yet surgical errors have often been managed with a focus on the individual surgeon, with an approach of ‘name, blame, shame and retrain’ [7].

In a high-reliability organisation (HRO), safety and quality (SQ) is an organisational priority, and all workforce members are engaged, continuously learning and improving their work. To build organisational capacity for SQ work, some healthcare organisations have developed capacity-building framework with a specific focus on patient safety and defined quality outcome measures, as part of an organisational strategy towards HRO [8].

A framework considering organisation-wide competencies for SQ includes all staff and faculty and is integrated into a broader organisation-wide operating management system for continuously improving quality. Achieving safe, high-quality care within this framework, is directly related to healthcare workforce training. In an article by Reason, he evaluated the cause of errors in healthcare utilising a system’s thinking approach. Describing the ‘Swiss cheese model’, Reason identified that healthcare errors are a combination of active failures related to individuals and also latent failures related to the overall healthcare system architecture [9]. Reason’s solution to improving patient safety was ‘systems thinking’ approach describing four increasing ‘barriers’ of defence, namely: (1) policy writing and training, (2) standardisation and simplification, (3) automation, and (4) improvement to devices and architecture.

In this paper, we assess and compare established practices in the aviation industry, that follow Reason’s principles, compared with current approaches to training in robotic surgery. Identifying what are the current similarities and also opportunities to evolve robotic training based on the evidence from established and proven aviation industry training practices. The review includes an assessment of how much of a ‘system’s thinking’ approach has been integrated into robotic surgery training and where the potential areas of improvement are for the future.

## Materials and methods

A systematic narrative review was performed with a comprehensive computerised search completed using PubMed and Medline databases. We systematically searched using subject headings including ‘robot-assisted surgery training’, ‘robotic surgery training’, ‘curriculum development’ and ‘proficiency-based training’, ‘pilot simulation training’, ‘aviation training’, and ‘pilot curriculum’.

Articles of interest included reports comparing aviation training with healthcare, prospective studies on the impact of robotic simulation training, robotic training curriculum development with validation and systematic reviews on robotic training published between July 2000, when the first robotic systems received FDA approval in the US [10]—and May 2019. Other significant studies cited in the reference list of selected papers were evaluated, as well as studies of interest published before the systematic search.

Two reviewers independently selected papers for detailed review evaluating the abstract and, if necessary, the full-text manuscript. Potential discrepancies were resolved by open discussion.

### Findings

The electronic search yielded a total of 253 potential articles. Following abstract review, 104 were critically reviewed for evidence synthesis. Overall, the quality of available studies was found to be low. Available evidence consists largely of expert opinion, consensus statements and small qualitative studies. There were no publications identified that focused on international standards for regulation within robotic-assisted surgery training. The review identified that there are many areas in robotic surgery and aviation training that share common approaches. Both have baseline evaluations, e-learning and simulation-based training and there is an increasing focus on non-technical skills training in robotic surgery [11]. Both industries also use checklists and automation to assess performance in real time. There was found to be significant time delays between the introduction of training initiatives in surgical training compared to the aviation industry. In Table 1, we have summarised the areas of commonality, categorising them under the headings of Reason’s ‘barriers’ to patient safety.

**Table 1:** Summary of initiative from aviation that have been replicated in surgical training

Description (level of barrier)	Airline industry	Healthcare system	Approximate delay to initiation (years)
Policy writing training (level 1)	The first checklist 1920	The WHO checklist 2008 [12]	88
Standardisation and simplification (level 2)	The first pilot license 1927	The first validated curriculum 2015 [13]	88
Simulation (level 2) (VR simulation level 3)	The first flight simulator 1920	The first VR simulation system 1993 [5]	73
Automation (level 3)	The first Black box 1953	VR simulation 1993 [5] Automated performance metrics [14] OR Black box 2018 [15]	65
Better devices and architecture (level 4)	The first control tower 1920	The first telementoring service 2001 [16]	81

### Checklists

Aviation checklists were introduced in 1920. Aviation checklists are divided into subsections with defined tasks that focus on critical segments such as takeoff, approach, and landing. Similar to the approach of the World Health Organisation (WHO), checklist which focuses on defined significant time points to complete the checklist, i.e., before induction, before skin incision and before the patient leaves the OR. Checklists have been introduced relatively recently into healthcare with the WHO ‘Surgical safety’ checklist, launched on June 25 2008 [12]. The implementation of the WHO checklist into the operating theatres was shown to result in a significant decrease in peri-operative mortality from 1.5 to 0.7% and inpatient complications from 11.0 to 7.0% [17].

### Training curriculum development

The first validated robotic training curriculum was published in 2015 [13]. It consists of various stages that are progressively completed with the trainee required to successfully pass each stage before progressing to the next, including e-learning, baseline evaluation, OR observation and first assistant training. Followed by a specialist attachment, such as a 'fellowship' at a high volume centre of expertise, with modular training, progressing to full procedure training and final assessment [13]. There is increasing awareness of the importance and benefits of standardised robotic surgery training and multiple specialist curricula have recently been developed by international panels of experts [18]. We are also seeing publications on guidance for procedural training [19].

E-learning is well established in the aviation industry. There is increased awareness of the benefits of e-learning in healthcare, in both increasing access to learning and driving standardisation in curricula content. There is also growing evidence on the effectiveness of e-learning on clinician performance and patient outcomes [20].

Non-technical skills training, error identification and crisis management have been key elements of aviation training for decades and there is evidence of increasing awareness and adoption of these important areas of robotic training [11, 21]. However, it is recognised that often robotic training curriculums currently prioritise technical skills training and lack training in the area of non-technical skills [22].

### Simulation training

Simulation can be defined as “a technique to replace or amplify real experience with or without guidance, often immersive in nature, that evokes or replicates aspects of the real world in a fully interactive manner” [15]. The first flight simulator was built in 1929 by Edwin Link. Driven by the cost of aircrafts and the financial and moral obligations to preserve human lives of both employees and customers, the airline industry has invested heavily in developing and standardising VR simulation training. Modern aviation simulators follow internationally agreed standards, are of high fidelity and can be utilised to train pilots in both standard and extreme conditions.

The history of surgical simulation can be traced back more than 2500 years when leaf and clay models were used to conceptualise nasal reconstruction with a forehead flap [23]. The current use of robotic surgery simulators allows trainees to develop the basic surgical skills outside of the operating theatre and enables better patient safety and standards of care [16]. VR simulation modules are now routinely used in the baseline evaluation of trainees [13]. We are also seeing the first examples of full procedure training in VR simulation. The efficacy of VR simulation is currently limited by the lack of comparative studies, standardisation of validation and high costs of development and purchase price [24]. Due to these current issues with VR simulation other forms of simulation are utilised in robotic training curricula. Table 2 summarises the current advantages and disadvantages of various simulation models.

**Table 2:** Summary of simulation models currently used in surgical training

Model	Strengths	Weaknesses
Task deconstruction models	Address metrics and are cost effective, e.g., Chicken gizzard model for vesico-urethral anastomosis [25]	Limited development to comprehensively address metrics, benchmarks and error management
Porcine model	Flexible training model for tissue handling	Expensive Not human anatomy No human pathology Limited accessibility
Canine cadaver model	Flexible training model for tissue handling	Not human anatomy No human pathology Limited accessibility
Human cadaver model	Flexible training model	Expensive Lacks human pathology and does not bleed Limited accessibility
3D printed models	Flexible training model Can incorporates pathology and vascularisation Increasingly realistic tissue handling Can incorporate metrics and benchmarks [26]	Currently, high development costs (lowered if printed casts rather than printed models) Models that address specific defined metrics need to be developed
VR simulation	Advanced procedural training models available (e.g., RARP, RAH)	Current scope/range/image quality limited
AR simulation	Potential to develop [27]	Limited development

### Air traffic control towers and telementoring services

Another area of support and enhanced feedback for pilots is the air traffic control tower. Telementorship programs have aimed to replicate this increased accessibility to expertise [28]. In 2001, the world’s first national telesurgery initiative was launched in Canada with the goal of disseminating expertise from large tertiary hospitals to remote and rural medical centres [28–30]. The Centre for Minimal Access Surgery group led by Dr. Mehran Anvari, located at McMaster University and St. Joseph’s Hospital in Ontario, Canada, trained surgeons through telementoring, utilising a dedicated Virtual Private Network (VPN) [29, 30]. Studies confirmed benefits to training and patient outcomes [31].

### The black box and automated performance metrics

The first ‘black box’ to record inflight data was first installed in 1953. This approach to automatically collect data from multiple recording devices to aid analysis and learning has recently been employed with the instillation of an operating room ‘Black Box’. The study identified ‘frequent intraoperative errors and events, variations in surgeon’s technical skills and a high amount of environmental distractions’, using the OR Black Box [32].

In a separate study, using a novel recorder of data taken directly from the da Vinci^®^ Surgical System (“dVLogger”), Hung et al. published evidence that objective surgeon performance metrics can be captured. Showing construct and concurrent validation of automated performance metrics (APMs) during robotic surgery [33].This study further highlights the potential of collecting automated data during robotic surgery.

## Discussion

Historically, many initiatives employed in aviation to improve training, data performance collection and enhanced feedback of performance have been replicated in surgical training. Often these occur many decades after evidence for these approaches has shown clear benefits to safety and quality in aviation (Table 1).

The aviation industry has successfully navigated the hurdles to integrated training and data collection with international agreement on standards and high-stake tests that result in quality assurance. Standardised robotic surgery curricula have been shown to be beneficial in both delivering education and identifying trainees who have not reached the pre-requisite skillets [6]. However, surgical curricula do not currently have the same levels of regulation or international standards compared to aviation. To achieve standards that are adopted internationally requires consensus on curricula content and structure. As the role of robotic-assisted (computer-assisted) surgery continues to expand, there are increasing opportunities to replicate the aviation industry technology-driven approaches. There is also increased need for this. As new robotic platforms enter the clinical scene, the development of standardised and validated training programs is increasingly important. Curricula development is a crucial step in the global standardisation of training, accreditation and certification of surgeons for robotic surgical procedures.

Further work is needed in the development, validation and implementation of these programmes to reduce variability within training [24]. The integration of validated VR simulation devices will likely aid the development of international standards [34]. Another highly successful approach to drive training standards, employed in aviation, is the use of qualified and regulated instructors. Train the trainer initiatives in robotic surgery have adopted this principle and can similarly drive standardisation with a ‘top down’ approach [35]. There is also increasing focus in train-the-trainer courses to recognise objective outcome metrics that will enable proficiency-based progression training [3].

From Reason’s defined four barriers to increasingly improving patient safety, we can see good evidence for the implementation of robotic training and policies to improve quality and safety such as the WHO checklist (1st level). There is increasing adoption of standardised robotic training curricula (2nd level). The third level comes from automation and in training we see evidence for this in VR simulation training modules with automated scoring systems and recently with automated performance metrics [33] and initiative such as the OR Black Box [32]. An advantage of VR simulator training over other simulation models is their ability to create objective performance data. Real-time feedback is provided, and results can be compared with validated data [24]. VR simulation training can, therefore, be practiced independently after suitable instruction. There are a number of robotic surgery training curricula developed in recent years which successfully include simulation training [13]. Both basic training and advanced procedural VR simulation training should be incorporated into robotic training curricula to shorten the learning curve without compromising patient safety. To enable this, we need cost-effective, high-fidelity, validated simulators to become incorporated into standardised, validated robot-assisted surgery training curriculum [34]. The development of more sophisticated training simulators that can accurately assess surgeon’s performance with objective metrics as well as automated collection of performance metrics (Hung et al.) will ultimately evolve current training curricula and enable international credentialing standards, as they have in aviation.

Reason’s 4th level barrier is improvement to devices and system architecture. There are similarities between control towers in aviation and telementoring services in surgery. Connecting training centres to educational hubs and telementoring services will result in robotic network development [36]. The potential and effectiveness of telementoring as an educational tool has been demonstrated, but the potential benefits of integration of telementoring and APMs into robotic training curricula to improve training system architecture have not yet been realised. This is partly due to issues related to setup costs and potential legal, data collection and ethical issues. Robotic network development would further enable automated data collection and comparison of outcomes. In the future, APMs could also result in device improvement with real-time alerts given by the surgical devices to help prevent surgical errors and events in the OR.

## Conclusions

Robotic surgeons and pilot roles have many fundamental similarities. Both work with expensive and complex technology requiring high levels of skills, within working environments that have high physiological and psychological stress levels. Historically many initiatives in aviation training have been replicated in surgical training, but there remain considerable differences in regulation between healthcare and aviation. Better understanding of the impacts of training and the ability to accurately and reliably measure surgical performance with objective metrics, will enable systems thinking in robotic surgery training. Adopting established and proven aviation methods of assessment and regulation could help robotic surgical training become more efficient, more effective and ultimately safer.
